# Research progress of nano selenium in the treatment of oxidative stress injury during hepatic ischemia-reperfusion injury

**DOI:** 10.3389/fphar.2022.1103483

**Published:** 2023-01-04

**Authors:** Xin Deng, Peng Ouyang, Wei Xu, Erhua Yang, Zhen Bao, Yijing Wu, Jin Gong, Jinghua Pan

**Affiliations:** Division 2, Gastrointestinal Surgery, First Affiliated Hospital of Jinan University, Guangzhou, China

**Keywords:** HIRI, oxidative stress, reactive oxygen species, nano selenium, antioxidant

## Abstract

Hepatic ischemia-reperfusion injury (HIRI) is an additional injury to ischemic tissue after hepatic revascularization, and its pathological mechanism is complex. HIRI is not only involved in the molecular targets that mediate cell death, such as ion channel activation, abnormal protease activation and mitochondrial dysfunction, but also related to the down-regulation of endogenous protective signals. As a by-product of normal aerobic metabolism, reactive oxygen species (ROS) act as a multi effect physiological signal factor at low concentration. However, liver ischemia-reperfusion will lead to excessive ROS accumulation, destroy redox homeostasis, lead to oxidative stress, cause cell death through a variety of mechanisms, and drive the further damage of ischemic liver. Recent studies have found that the antioxidant treatment of nano selenium can reduce the excessive production of ROS and play a potential protective role in reducing HIRI. This paper reviews the molecular mechanism of the antioxidant effect of nano selenium for the prevention and treatment of HIRI, in order to provide further experimental basis for the clinical prevention and treatment of HIRI.

## Introduction

Hepatic ischemia-reperfusion injury (HIRI) refers to the recovery of hepatic perfusion blood flow after a period of ischemic liver injury, which leads to the further aggravation of the original liver injury. Its occurrence is an important reason for postoperative liver function injury, liver failure and even death. At present, it is considered that the role of calcium overload is closely related to the destruction of the structure and function of neutrophils. HIRI can be divided into two stages: ischemia and reperfusion. In the ischemic stage, ATP concentration in cells decreases, leading to Na/K pump failure, cell edema and increased cytoplasmic calcium concentration, which will lead to cell damage. At the early stage of reperfusion (i.e., within 2 h after reperfusion), Kupffer cells release proinflammatory mediators (TNF-α, IL-6, IL-1 and arachidonic acid) and reactive oxygen species (ROS); In the late stage of reperfusion (i.e., 6–48 h after reperfusion), neutrophil mediated inflammatory reaction occurs. Complement factors, chemokines and Cytokines recruit neutrophils into the liver and destroy the liver cells by releasing ROS or proteases.

Among them, a crucial role in HIRI is played by oxidative stress. Excessive ROS can lead to calcium overload, apoptosis, up regulation of cytokines and changes of DNA and protein (Cadenas, 2018), which can aggravate liver ischemia-reperfusion injury. Therefore, the effective elimination of excess oxygen free radicals or ROS may provide a theoretical basis and therapeutic target for reducing HIRI.

Because the molecular mechanism involved in ischemia-reperfusion injury is very complex and the correlation between various pathways is very close, this leads to the limited effect of a single intervention factor on liver ischemia-reperfusion injury, and can not comprehensively and fully avoid the occurrence of liver ischemia-reperfusion injury. Drugs for HIRI treatment should meet multiple treatment indexes at the same time, so as to give better play to the curative effect of drug treatment. It has been reported that nano selenium can play an antioxidant role by directly scavenging reactive oxygen species, enhancing the antioxidant capacity of enzymatic antioxidant system and inhibiting cell apoptosis. Because nano selenium can play an important role in preventing and protecting HIRI caused by oxidative stress, this paper reviews the progress of nano selenium in regulating oxidative stress in HIRI, in order to provide reference for clinical prevention of HIRI.

## Oxidative stress and reactive oxygen species

When subjected to various harmful stimuli, the human body overproduces highly reactive molecules such as reactive oxygen species (ROS) and reactive nitrogen (RNS), so that the degree of oxidation exceeds the removal of oxidants, and the balance between the oxidation system and the antioxidant system is broken, resulting in tissue damage, which is Oxidative Stress. ROS mainly refers to hydroxyl radicals (•OH), superoxide anions (•O_2-_), and hydrogen peroxide (H_2_O_2_). It is worth noting that ROS, as a second messenger, can be used as a physiological signal factor in the normal process of cell growth, migration and death when it exists in low concentration (Venditti and Di Meo, 2020). For example, by regulating inflammatory molecules and cytokines, ROS can acts as a secondary messenger (Blaser et al., 2016). However, during liver ischemia-reperfusion, it will also promote the complex pathological processes of succinate accumulation, lysosomal crosstalk, endoplasmic reticulum stress and oxidase up regulation, resulting in the excessive accumulation of ROS, and aggravate the occurrence and development of hepatocyte injury through MPTP opening, excessive autophagy, inflammatory body activation and immune response activation, affecting the prognosis of patients with reperfusion (Jun et al., 2019; Xu et al., 2019).

**FIGURE 1:**
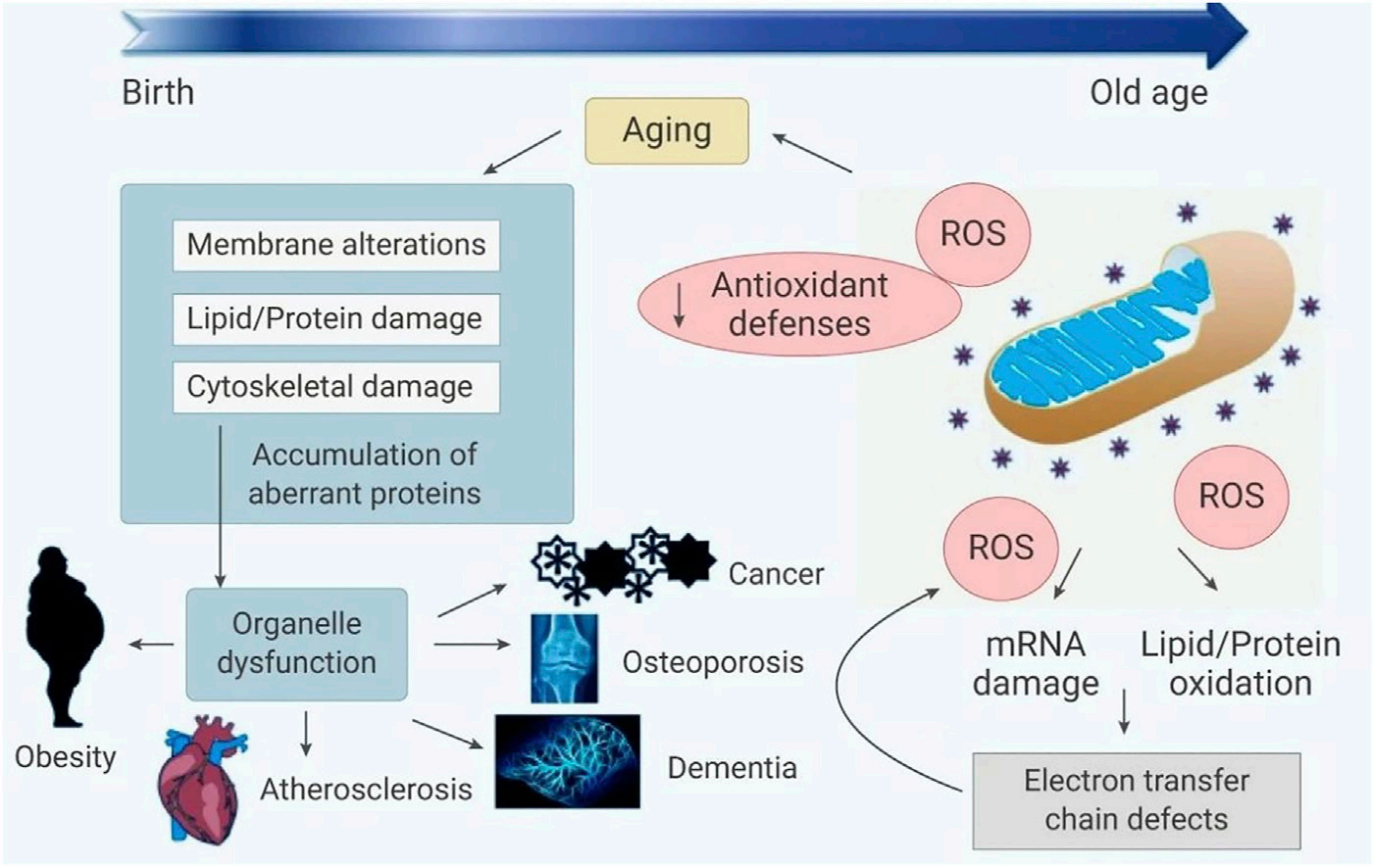
The relationship between oxidative stress and disease.

In general, there are a number of substances in cells that are susceptible to ROS. ROS can not only cause cell damage, but also produce harmful byproducts such as lipid oxides and lipid peroxides. On the other hand, excessive ROS can also directly cause protein and DNA damage, block enzyme activity, and even lead to cancer (Kumar et al., 2008). The imbalance of ROS production and elimination is considered to be related to oxidative stress, leading to mitochondrial dysfunction. Oxidative stress directly or indirectly leads to various diseases (Andersen, 2004; Barnham et al., 2004; Houstis et al., 2006; Ishikawa et al., 2008), including stroke (Sarmah et al., 2019), sepsis (Hoetzenecker et al., 2012), diabetes (Liang et al., 2018), hypertension (Touyz, 2003), neurodegenerative diseases (Radi et al., 2014), inflammation (Blaser et al., 2016) and cancer (Schumacker, 2015), (as shown in Figure 1). Therefore, by regulating ROS production or neutralizing ROS, it is possible to prevent and treat diseases related to oxidative stress (Zhao et al., 2020).

## Mechanism of HIRI induced by oxidative stress

### The openning of mitochondrial permeability transition pore (mPTP)

**FIGURE 2:**
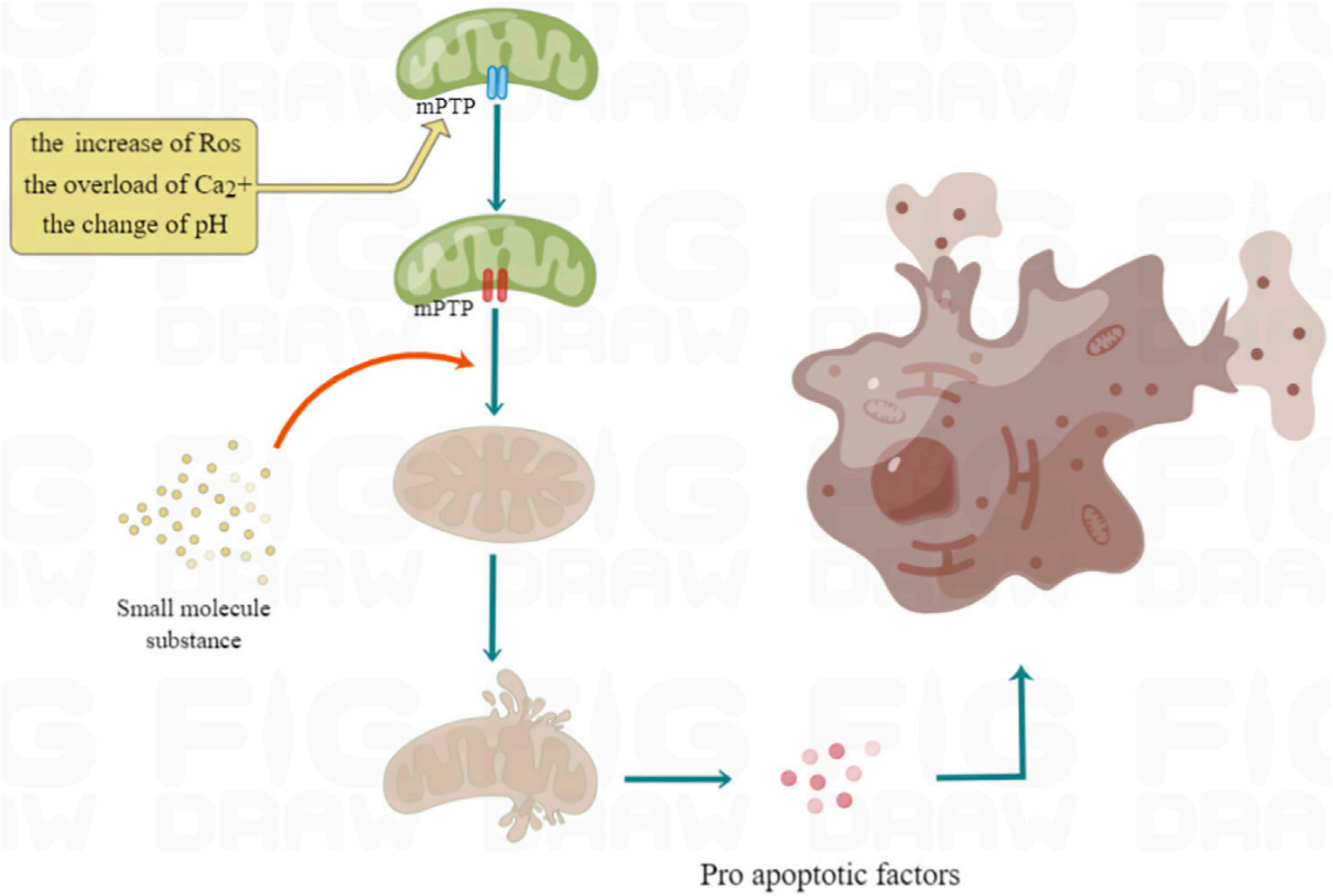
Mechanism of excessive opening of mPTP leading to cell death. The overload of Ca2+, ROS increase and pH change will all lead to the opening of mPTP. These factors can prolong the opening time of mPTP by activating the important component of mPTP, Cyp D, so as to make the electrochemical proton gradient collapse, dissolve coupling with oxidized phosphoric acid, stop ATP synthesis, and then cause cell necrosis.

The mitochondrial inner and outer membranes contain mPTP, which is a multiprotein complex. It is a non-specific channel between mitochondrial matrix and cytoplasm to maintain calcium homeostasis, regulate oxidative stress signals, cause protein translocation and other signal transduction or material transfer (Cadenas, 2018). First of all, the opening of MPTP will make the mitochondrial inner membrane non-selective penetration of molecules with molecular weight less than 1500, collapse the mitochondrial membrane potential and decouple oxidative phosphate, resulting in ATP consumption and cell death. Secondly, it can lead to mitochondrial respiratory interruption, matrix swelling and membrane rupture, and deposit Pro apoptotic factors from the membrane gap to the cytoplasm, resulting in apoptotic cell death (Ong et al., 2015). Therefore, prolonging the opening time of MPTP usually changes the role of mitochondria from supporting cell survival to actively inducing apoptosis and necrotic cell death, which has become the main event of cell intrinsic death pathway (Xu et al., 2019), (as shown in Figure 2).

### Excessive autophagy

**FIGURE 3:**
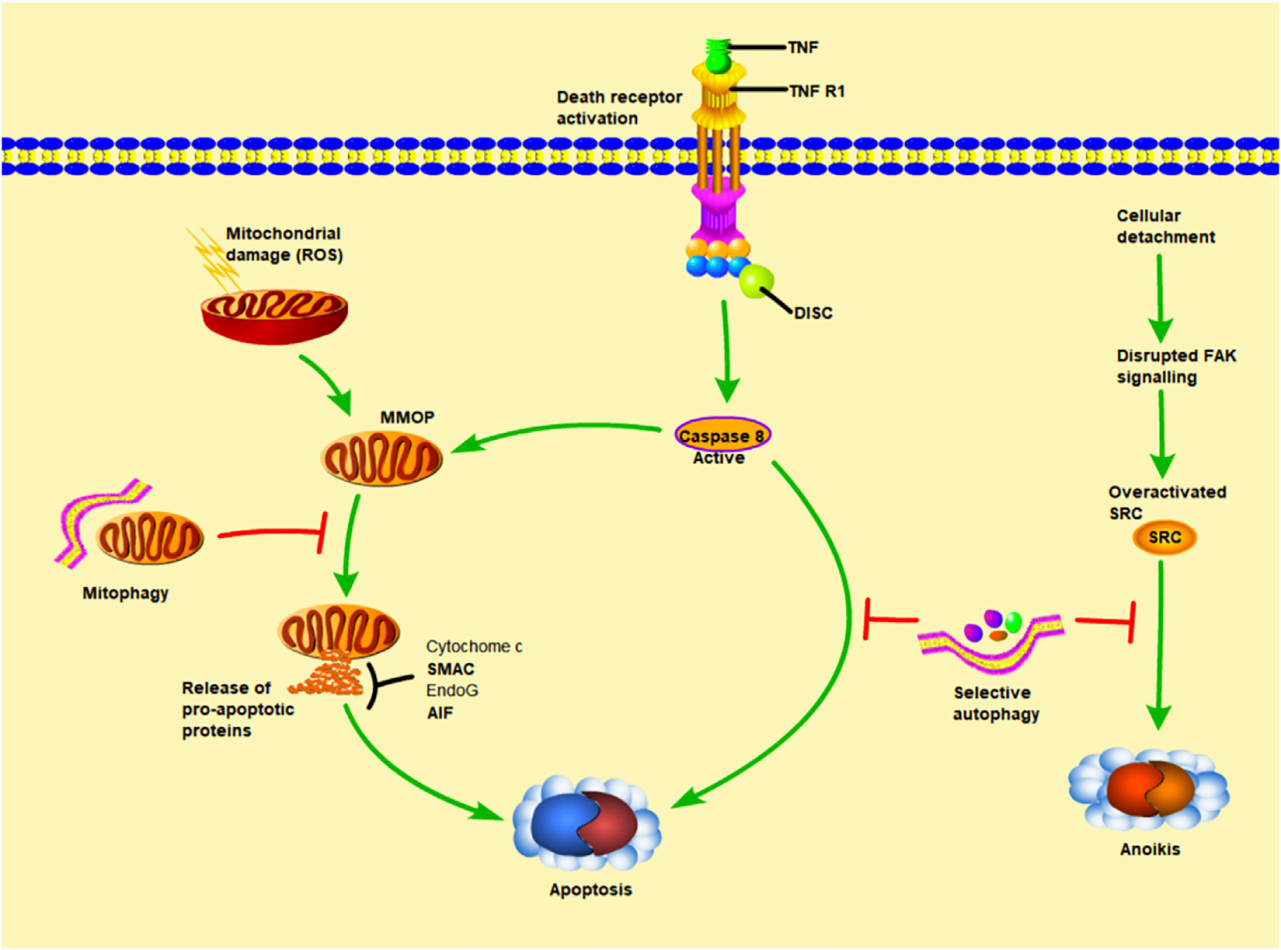
The relationship between autophagy and apoptosis. MOMP (mitochondrial outer membrane permeability) leads to the release of cytochrome c (cytochrome c), AIF (apoptosis inducing factor), EndoG (endonuclease G). Among them, cytochrome c can promote caspase-9 and APAF1 (apoptosis protease activating factor 1) to combine into apoptosis complex, thereby inducing downstream apoptosis. The other way that can independently promote apoptosis is LMP (lysosomal membrane permeability), which stimulates the release of cathepsin into the cytoplasm. Cathepsin can also promote MOMP and strengthen the apoptosis pathway other than its own. Under normal conditions, autophagy maintains intracellular homeostasis by removing damaged proteins and aging organelles. However, during liver ischemia-reperfusion, a large amount of autophagy will lead to the accumulation of autophages and abnormal degradation of proteins and organelles, thus accelerating this process.

By removing damaged proteins and aging organelles, basal level autophagy maintains intracellular homeostasis. However, autophagy does not always have a protective effect during liver ischemia-reperfusion. A high amount of autophagy will result in an accumulation of autophagosomes and abnormal degradation of proteins and organelles (Liu et al., 2018). Previous studies have shown that oxidative burst during reperfusion can promote p53/hepatocyte signal transduction, induce microtubule associated protein 1 light chain 3 lipidation, up regulate autophagy flux and the expression of autophagy key protein Beclin1, greatly improve autophagy activity, lead to hepatocyte death and accelerate ischemic injury (Liu et al., 2018), (as shown in Figure 3).

### Inflammatory corpuscle activation

**FIGURE 4:**
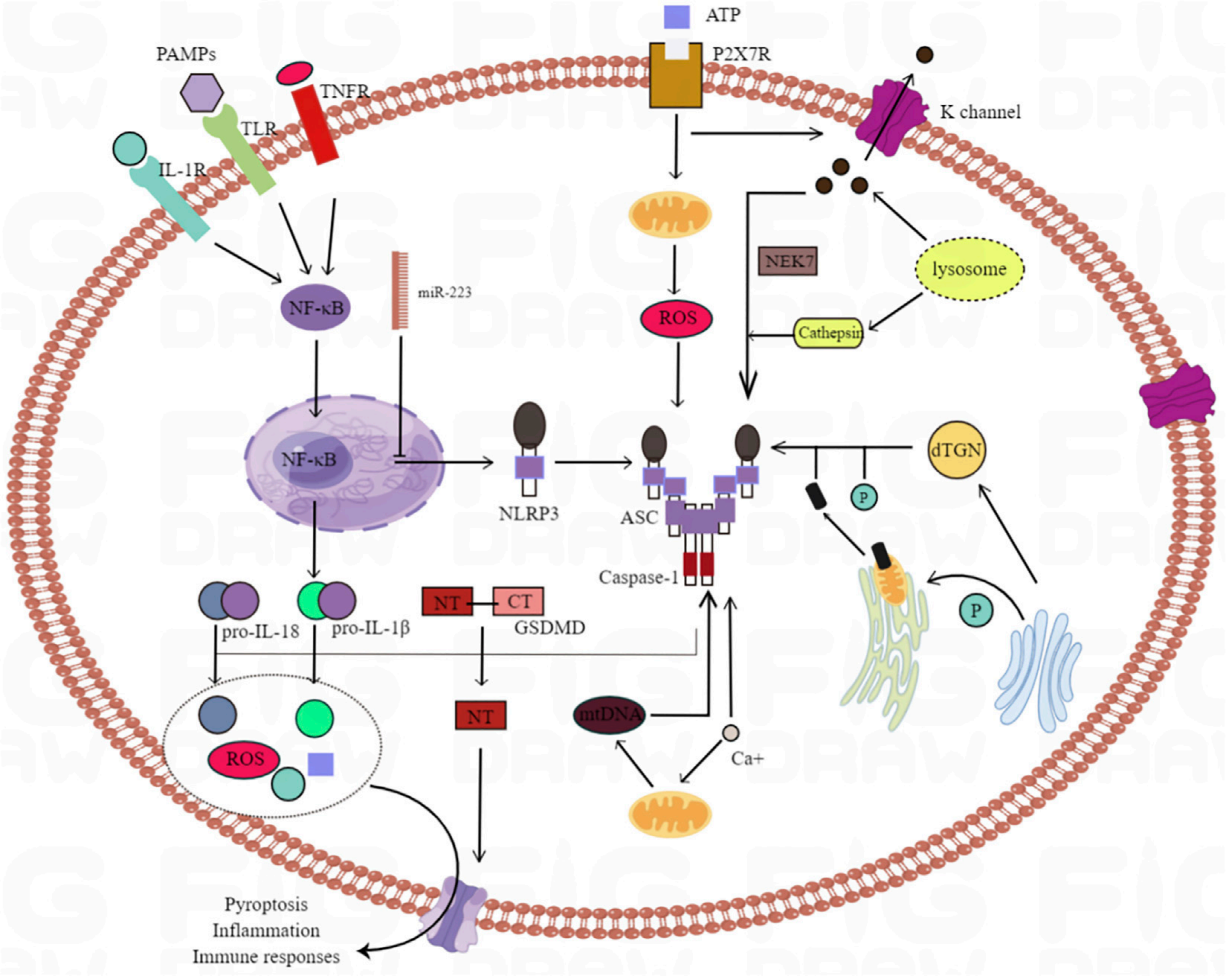
The mechanisms underlying NLRP3 inflammasome activation. There are many trigger factors for the activation of NLRP3 inflammatory bodies, including K^+^ efflux, Ca^2+^ mobilization, endoplasmic reticulum stress, lysosomal leakage and mitochondrial dysfunction. NLRP3 activation is also triggered by several mechanisms involved in Golgi vesicle transport of the Golgi apparatus and endoplasmic reticulum. Dispersed TGN (trans Golgi network) provides a scaffold for the formation of NLRP3 lacrimal dots, and promotes the assembly and activation of NLRP3 inflammatory bodies; PtdIns4P as the binding site. Activation of NLRP3 inflammatory bodies can induce GSDMD (gasdermin D) and pre IL ‐ 1 β And pre IL ‐ 18. NT‐GSDMD (N terminal ‐ GSDMD) forms a hole on the cell membrane to induce the pyrolysis and release of intracellular factors.

Inflammasome is a multi protein complex, including caspase-1, apoptosis related spot like protein and NLRP3. As an integral part of the innate immune system, the activation of inflammatory bodies induces caspase-1 to cleave the desqualin D protein, causing the cells to swell until the membrane integrity is lost, and promotes interleukin (IL) - 1β, The maturation and secretion of IL-18 and the leakage of cell contents lead to a pro-inflammatory form of programmed cell death called focal death (Dai et al., 2020). In recent years, two studies have explained two different mechanisms of NLRP3 activation through experiments *in vivo* and *in vitro*: the former is activated by nuclear factors at the transcriptional level κB-driven, nuclear factor in ischemia-reperfusion injury κB is activated by toll like receptor (TLR) signaling pathway in myeloid differentiation factor 88 dependent pathway, and then stimulates IL-1β, IL-18 expression (Dai et al., 2020). The latter is induced at the post transcriptional level, including the activation of NLRP3, driven by many activators, such as excessive ROS in ischemia-reperfusion injury (Jun et al., 2019). Because the activation of NLRP3 inflammasome is very important in HIRI, some scholars have proposed to inhibit NF-κB and acanthin D protein can reduce inflammatory body induced cell death (Liu et al., 2017). At the same time, previous studies have shown that both mitochondria and NOx derived ROS can up regulate thioredoxin interacting proteins, thus mediating the activation of NLRP3 inflammatory bodies, leading to inflammation and scorch death of cells (Han et al., 2018a; Jun et al., 2019). By intraperitoneal injection of antioxidant drugs into the animal model, the interaction between thioredoxin interacting protein and NLRP3 can be blocked, so as to inhibit the activation of NLRP3 inflammatory body and IL-1β (Han et al., 2018a), (as shown in Figure 4).

### Immune response activation

It is well known that ischemia-reperfusion can initiate immune response and then activate acute inflammatory response, which is characterized by immune cell recruitment and activation related to innate and adaptive immune system (Fan et al., 2019). There is evidence that ischemic hepatocytes can promote innate immune response in addition to serving as target organs (Oyama et al., 2004). When damaged hepatocytes release injury-related molecular patterns and interact with pattern recognition receptors, this immune response is activated, and ROS, as an important injury related molecule, plays a driving role in the whole process (Vilahur and Badimon, 2014). Among many pattern recognition receptors, TLR has attracted extensive attention, among which TLR4 plays an important role in liver diseases. In addition, macrophage phenotypic polarization is also an important part of immune response activation. Excess ROS has been shown to cause macrophages to polarize to the M1 phenotype, possibly due to activation of pattern-recognition receptors such as C-type lectin receptors, TLRs, and nod-like receptors (Fan et al., 2019). When macrophages polarize to M1 phenotype, they can directly release IL-23, IL-1 β and tumor necrosis factor- α, up regulate the secretion of chemokine ligand 1 and granulocyte colony stimulating factor, and indirectly mediate the infiltration of neutrophils and the production of proteolytic enzymes. The recruitment of neutrophils will further aggravate the production of ROS, cause a vicious circle and aggravate liver injury (Formentini et al., 2017; Fan et al., 2019). Formentini et al. (Formentini et al., 2017) found that targeted inhibition of mtROS can polarize macrophages from M1 phenotype to M2 phenotype with anti-inflammatory effect, so as to reduce cell damage. The above data show that immune response has a great impact on HIRI.

## The main source of ROS in HIRI

### O_2_^−^ and H_2_O_2_ produced by mitochondria

**FIGURE 5:**
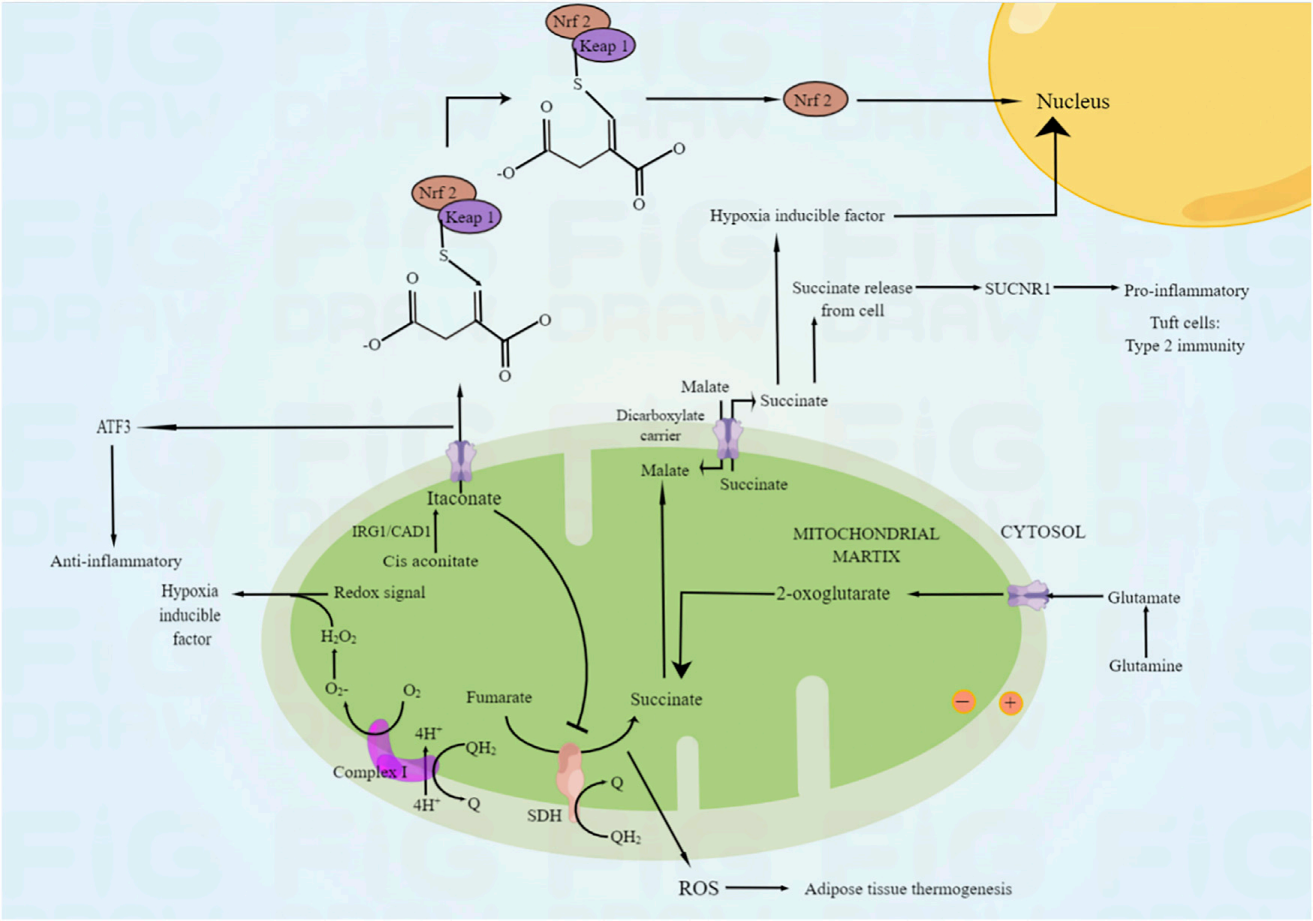
The mechanism of ROS production in mitochondria. Under the action of inflammation and hypoxia signal, the reverse of succinate dehydrogenase will lead to the accumulation of ischemic succinate, leading to the decomposition of purine nucleotides and the reverse of some malic acid, leading to the overflow of fumaric acid. After reperfusion, succinic acid was rapidly re oxidized by succinate dehydrogenase through reverse electron transport of mitochondrial complex I, and a large amount of ROS was produced.

Mitochondria are the main sites of ROS production in many tissues and organs (Cadenas, 2018). During the period of ischemia and hypoxia, ATP content decreased and Ca2+concentration increased, which caused damage to the mitochondrial function, leading to the impairment of the activity of respiratory chain reactivators, the decline of the efficiency of respiratory chain to transfer electrons, and the inability to generate enough electrons, which increased the generation of free radicals; A large amount of ROS will be produced during oxygen supply by reperfusion, and ROS can be produced by respiratory chain complexes I, II and III at this time. There are 11 different mitochondrial sites that catabolize substrates and transport electrons to produce O_2·-_ and H_2_O_2_ (Cadenas, 2018). It has been suggested that mitochondrial reactive oxygen species (mtROS) is a non-specific product of ischemia-reperfusion-induced dysfunction of the respiratory chain (Touyz, 2003); However, Chouchani et al. (Radi et al., 2014) found that during tissue ischemia, under the action of inflammatory and hypoxia signals, it is a common metabolic feature to accumulate succinic acid in the citric acid cycle, and mtROS is produced during reperfusion, resulting in cell damage. This study confirmed for the first time that ischemic succinate accumulation occurs when succinate dehydrogenase reverses, resulting in fumarate overflow due to purine nucleotide decomposition and partial malate reversal. After reperfusion, through reverse electron transport of mitochondrial complex I, succinate is rapidly reoxidized by succinate dehydrogenase and produces a large number of ROS (Radi et al., 2014). At the same time, the increase of ROS in damaged mitochondria will promote the production of ROS in adjacent mitochondria, lead to the further up regulation of ROS level in cells, and trigger the positive feedback mechanism of ROS induced ROS release (Schumacker, 2015), (as shown in Figure 5).

### OH· produced by lysosome

Lysosomes are responsible for the regulation of damaged or excess structures and maintain autophagic degradation. This compartment is rich in loose redox active iron as a result of degrading a variety of iron-containing macromolecules, which can react with a small amount of H_2_O_2_ escaping from mitochondria to produce OH · (Li et al., 2016). Most pathological processes, including myocardial ischemia-reperfusion, may cause oxidative stress to be activated in mitochondria, and then produce a large amount of H_2_O_2_. Amplify the crosstalk between mitochondria and lysosomes, resulting in the continuous accumulation of OH · in lysosomes, promoting the peroxidation and permeability of lysosomal membrane, leading to the loss of stability, releasing a large amount of cathepsin, and inducing cell apoptosis or necrosis (Ullio et al., 2015; Li et al., 2016).

### ROS generated by endoplasmic reticulum

Endoplasmic reticulum is an important place for efficient synthesis and folding of proteins. In the process of oxidative protein folding, the formation of disulfide bond is a main process. In the catalytic reaction of protein disulfide isomerase, each disulfide bond formed will produce an oxidation equivalent H_2_O_2_, which is the result of reoxidation of protein disulfide isomerase catalyzed by endoplasmic redox 1. As a by-product of protein folding, H_2_O_2_ can be utilized by glutathione peroxidase to reduce the oxidative load of endoplasmic reticulum (Sies and Jones, 2020). However, ischemia-reperfusion will increase unfolded protein response and misfolded protein accumulation, resulting in endoplasmic reticulum stress pathology. In this context, the redox balance of endoplasmic reticulum is unbalanced, and excess H_2_O_2_ cannot be removed and utilized (Zhu et al., 2018; Sies and Jones, 2020). Additionally, activation of unfolded protein response has been shown to increase ROS levels by affecting mitochondrial function (Zhu et al., 2018), which also proves that mtROS can be produced by a variety of pathological mechanisms.

### ROS produced by reduced nicotinamide adenine dinucleotide phosphate oxidase (NOX) and xanthine oxidase (XO)

There are seven subunits of NOX: NOX1, NOX2, NOX3, NOX4, NOX5, duox1 and Duox2. TBy catalyzing the electron transfer from reduced nicotinamide adenine dinucleotide phosphate to molecular oxygen, they produce H_2_O_2_ and O_2 ·_. Among them, Nox1 and NOX2 directly produce O_2_·, but NOX4 located in mitochondria, unlike other isomers, produces H_2_O_2_ through rapid disproportionation of O_2_· - and these three subunits have also been proved to be expressed in cardiomyocytes (Zhang et al., 2020). Braunersreuther et al. (Braunersreuther et al., 2013) found that by knocking out these NOx subunits in animal models, protein phosphorylation such as protein kinase B and extracellular regulated protein kinase increased, myocardial ROS decreased and infarct size decreased significantly. In addition, XO is a key enzyme in purine catabolism. It involves a variety of pathways in oxidative stress response (including nitric oxide and calcium signal transduction), which can bind excess electrons to electron receptors such as oxygen and produce a large amount of ROS (Zhu et al., 2018). It is found that XO acts on the downstream of NOx in ischemia-reperfusion injury, which means the existence of oxidase crosstalk. The first activated primary oxidase seems to be NOx, which can activate the downstream oxidase, such as XO or uncoupled endothelial nitric oxide synthase, and lead to the secondary generation of ROS (Zhang et al., 2020). Therefore, ischemia-reperfusion requires all kinds of oxidases.

## Antioxidant mechanism of nano selenium

**FIGURE 6:**
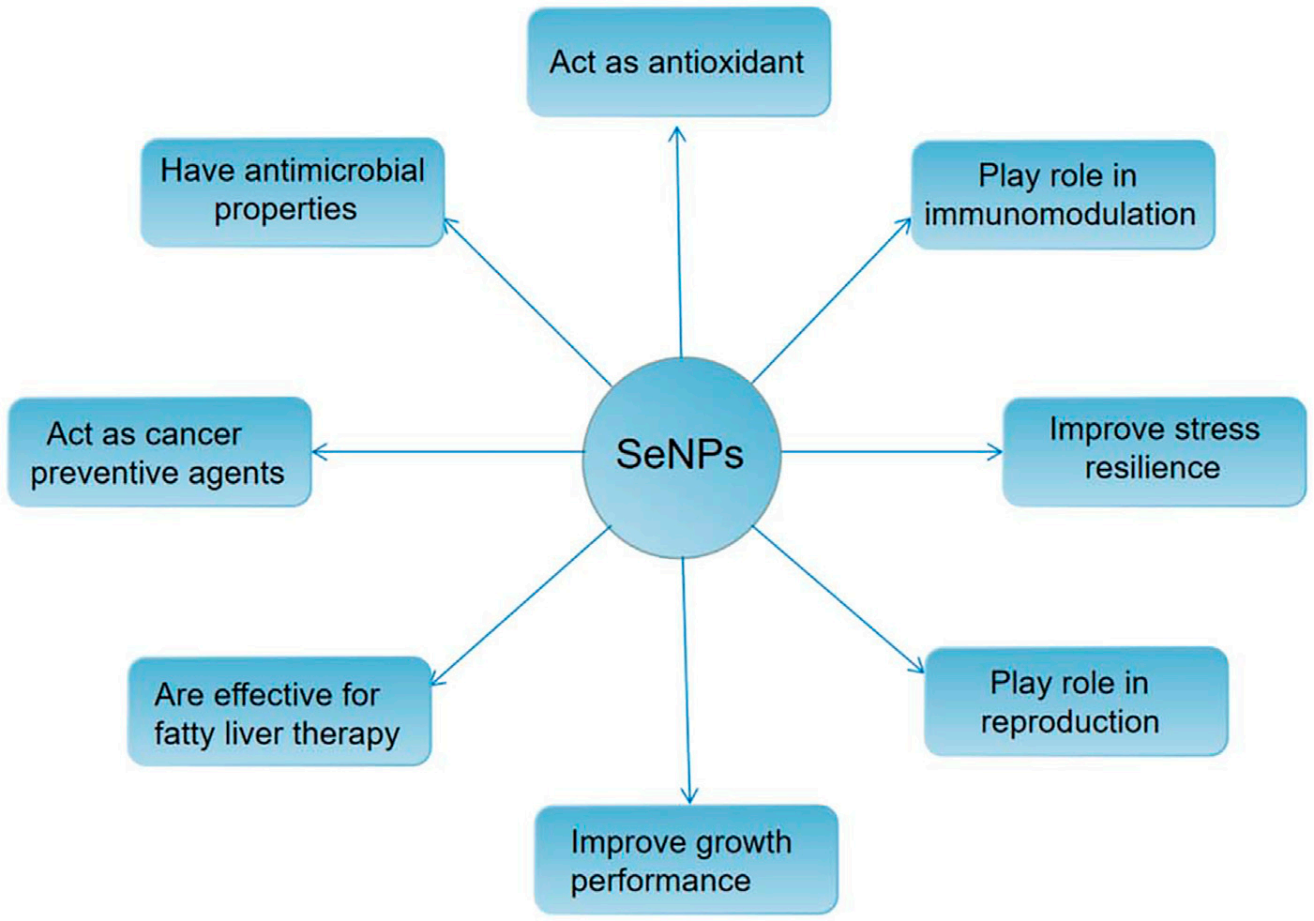
The application of nano selenium in medical field.

In human life activities, selenium (SE) plays an important role as one of the essential trace elements. It mainly exists in organic selenium in the body, including selenase, selenium ribonucleic acid, selenoprotein and other forms (Chung et al., 2020; Yuan et al., 2020). It is a variety of enzyme cofactors in the body, such as catalase (CAT), superoxide dismutase (SOD), glutathione peroxidase (GSH PX) and so on. By removing free radicals and other substances from cells, it has a direct or indirect effect. Besides protecting cells from oxidative stress and preventing cardiovascular disease, it also plays an important role in hypercholesterolemia and cancer (Yuan et al., 2020). At present, organic selenium and inorganic selenium compounds have been used as dietary supplements of selenium for several years, but due to their very narrow safety range, careless dosage is very easy to cause selenium poisoning (Fan et al., 2020); The red elemental selenium has low toxicity and certain biological activity, but its chemical stability is poor. It is easy to aggregate into an inactive state under rapid heating or room temperature (Mellinas et al., 2019). In 1997, Zhang Jinsong and others (Zhang et al., 2001) creatively applied nanotechnology to the preparation of red elemental selenium for the first time. They introduced bovine serum albumin (BSA) as a protective agent to successfully prepare nano selenium with stable structure and high biological activity. Compared with other valence organic selenium and inorganic selenium compounds, the red elemental selenium prepared by nanotechnology not only has higher bioavailability and better safety, but also has good antioxidant activity, which can significantly improve the abnormal oxidative stress state of cells (Bai et al., 2020), (as shown in Figure 6). Its action mechanism mainly includes the following points:

### Scavenging reactive oxygen species

Nano selenium can directly remove ROS. The nano selenium spherical particles modified by CS with different molecular weight have good scavenging ability to DPPH, ABTS and other free radicals; In a certain range, the inhibition intensity of ROS in skin and intestinal cells depends on the concentration of selenium in nano materials. The higher the concentration, the greater the inhibition intensity of ROS (Zhai et al., 2017). Huang et al. (Huang et al., 2003) made nano selenium from red elemental selenium nanoparticles and bovine serum albumin (BSA) and found that nano selenium can directly scavenge free radicals, such as DPPH, superoxide anion and singlet oxygen; *In vitro*, the ability of nano selenium with different particle sizes to directly scavenge free radicals is significantly different. The smaller the particle, the better the scavenging effect. Tong Chunyi et al. (Tong et al., 2006) detected the antioxidant activity of nano selenium by fluorescence method. The results showed that the scavenging effect of nano selenium on free radicals at low concentration was stronger than that of vitamin C and Na_2_SeO_3_; The maximum clearance concentration of nano selenium is about 1/3 of vitamin C and 1/5 of Na_2_SeO_3_; The semi clearance concentration of nano selenium is also lower than that of the other two antioxidants. It shows that nano selenium has good antioxidant effect, and has great advantages in dose. It is an efficient antioxidant.

### Enhance the antioxidant capacity of enzymatic antioxidant system

Both enzymatic and non-enzymatic antioxidant systems can clear ROS. Non-enzymatic antioxidant system is mainly divided into water-soluble antioxidants and fat soluble antioxidants; Enzymatic antioxidant system includes various antioxidant enzymes, such as SOD, CAT, GPX and TRX. SOD and cat are very important antioxidant enzymes *in vivo*. As a first line of defense against ROS, SOD converts superoxide free radicals into H_2_O_2_ and water; CAT further catalyzes H_2_O_2_ to produce water and oxygen (Hamza and Diab, 2020).

Nano selenium can enhance the antioxidant capacity of enzymatic antioxidant system *in vivo*. Loeschner et al. (Loeschner et al., 2014) found that nano selenium has a similar metabolic pathway to selenite in rats: the nano selenium absorbed into the body may be first transformed into H_2_Se, and then incorporated into the active sites of various selenoproteins such as GPX and TRX, so as to significantly improve the activity of selenoproteins and prevent oxidative damage to the body. The intake of nano selenium particles first leads to the reduction of human ROS level. Before apoptosis, it stimulates the transcription of various downstream antioxidant enzyme genes by activating nuclear transcription factor Nrf2, promotes the human body to clear ROS and protects the intestine from oxidative stress (Song et al., 2017).

### Inhibition of apoptosis

Nano selenium can play an antioxidant role by inhibiting apoptosis. By upregulating the expression of anti-apoptotic protein Bcl-2 and down-regulating the expression of apoptotic protein Bax, nanoselenium regulates mitochondrial membrane permeability and inhibits the release of pro-apoptotic proteins into the cell, including cytochrome C, thereby inhibiting the occurrence of apoptosis (Sadek et al., 2017). In addition, nano selenium can regulate p38 MAPK/ERK, nuclear transcription factor kappa B (NF-κB), ASK1/JNK, PI3-K/Akt/mTOR and other signaling pathways to inhibit apoptosis induced by oxidative stress.

## Functionalized modified nano selenium

Although the good antioxidant effect of selenium nanoparticles has attracted the attention of researchers, due to the specific intracellular function of an appropriate amount of ROS, while ordinary antioxidants will affect healthy tissues that have not suffered oxidative damage while providing intervention treatment. At the same time, researchers also found that some receptors, proteins or enzymes are abnormally high expressed in specific damaged cells, but not expressed or low expressed in normal tissues and organs. It is possible to use these expression differences to guide drugs to target cells, so as to improve effects and reduce side effects. Therefore, based on the above view, targeted antioxidants show great potential. For example, mitoxantrone methanesulfonate can combine with lipophilic triphenylphosphine to produce active antioxidant ubiquitin in mitochondria and prevent the production of mtROS in ischemia-reperfusion model (Han et al., 2018b).

In the application field of nano selenium, functional modified nano selenium is also a research hotspot in recent years. The nanoparticles modified by targeted polypeptide surface can bind to the receptor overexpressed by targeted cells, so as to achieve the purpose of specific drug function. In addition, the surface modified nanoparticles can also improve the absorption and efficacy of drugs by corresponding target cells. At present, many scientific research teams have conducted in-depth research on the functionalization of nano selenium and prepared a variety of functionalized nano selenium. For example, the study of Chen et al. (Rao et al., 2019) demonstrated that SeNPs@GM1/TMP inhibited p53 activation as well as MAPK pathways, thereby preventing mitochondrial dysfunction by preventing ROS overproduction, and can induce PC12 cell apoptosis by inhibiting DNA mediated p53 phosphorylation through the protective effect of t-BOOH. The results of Huang et al. (Liu et al., 2020) clearly demonstrate that L-G@SeNPs exhibits preferential aggregation in internal organs, especially the liver, while in contrast, DL- and d-counterparts evade liver uptake and undergo faster renal clearance. L-G@SeNPs by utilizing homologous adhesion between L-GSH and L-phospholipid membranes, the affinity with cell membranes is increased and extended to higher concentrations nearby, preventing oxidative damage of PA in INS-1 cells because PA weakens ROS and mitochondrial disruption D-G@SeNPs at higher levels.

## Summary

In conclusion, ROS mediated oxidative stress plays an important role in HIRI. Reducing the large amount of ROS produced during HIRI is of great significance to reduce HIRI. Nano selenium, as an antioxidant with high efficiency and low toxicity, shows a good application prospect in anti HIRI. Compared with other forms of selenium, nano selenium can not only act as a separate antioxidant drug, but also play an antioxidant role by directly scavenging reactive oxygen species, enhancing the antioxidant capacity of enzymatic antioxidant system and inhibiting cell apoptosis; It can also further improve the antioxidant capacity and targeting through functional modification, and play an important role in preventing and protecting HIRI caused by oxidative stress. With the continuous improvement of scientific and technological level and the deepening of research, a nano selenium drug with strong antioxidant capacity, good targeting and high stability may appear in the future, which can be used in the treatment of clinical HIRI.
